# Breakfast Skipping Increases the Risk of Coronary Heart Disease and Myocardial Infarction: Evidence From the Mendelian Randomization Study

**DOI:** 10.1002/fsn3.70998

**Published:** 2025-09-18

**Authors:** Si Cao, Youjie Zeng, Gong Chen

**Affiliations:** ^1^ Department of Anesthesiology Third Xiangya Hospital, Central South University Changsha Hunan China

**Keywords:** breakfast consumption, cardiovascular diseases, causal inference, Mendelian randomization, risk factors

## Abstract

Observational studies have suggested a potential association between breakfast skipping and the risk of coronary heart disease (CHD) and myocardial infarction (MI). Nevertheless, due to potential confounders and reverse causation, the causal relationship remains unclear. This study aims to comprehensively assess the causal effect of breakfast skipping on CHD and MI through a two‐sample Mendelian randomization (MR) analysis. Genome‐wide summary statistics of breakfast skipping, CHD, and MI were obtained from public databases. Instrument variables (IVs) proxying for breakfast skipping were screened based on strict (*p* < 5e−8) and lenient (*p* < 1e−5) thresholds. Quality control of IVs was performed using Cochran's *Q* test, MR‐Egger intercept test, and MR PRESSO global test. Inverse variance weighted (IVW) served as the primary MR method for assessing causal associations, while MR‐Egger, weighted median, and maximum likelihood were used as supplementary methods. The effect of breakfast skipping on CHD and MI in both CARDIoGRAMplusC4D and FinnGen cohorts was assessed, and the results were integrated using meta‐analysis. Quality control tests showed no heterogeneity or horizontal pleiotropy in IVs. After conducting a meta‐analysis of the MR results from the IVW method under strict IV screening thresholds, breakfast skipping significantly increases the risk of CHD [odds ratio (OR) = 1.358, *p* = 3.95E−02] and MI (OR = 1.449, *p* = 4.17E−02). Similarly, meta‐analysis of the IVW results under lenient IV screening thresholds also demonstrated the detrimental effects of breakfast skipping on CHD (OR = 1.198, *p* = 5.55E−03) and MI (OR = 1.378, *p* = 4.08E−05). Three other MR methods showed parallel results to IVW (OR > 1). Overall, the present study demonstrates that breakfast skipping significantly increases the risk of CHD and MI. Therefore, maintaining a high‐quality breakfast intake can be considered an essential primary prevention measure for CHD and MI.

## Graphical abstract

This study uses Mendelian Randomization to investigate the causal relationship between breakfast skipping and cardiovascular risk. The findings reveal that breakfast skipping significantly increases the risk of coronary heart disease and myocardial infarction. These results highlight the importance of breakfast as a preventive measure for heart health.

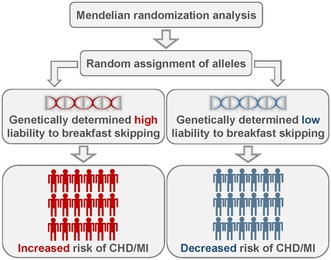

## 1 Introduction

Coronary Heart Disease (CHD) remains one of the leading causes of mortality globally, imposing a substantial public health burden and often manifesting as myocardial infarction (MI) or ischemic cardiomyopathy (Dalen et al. 2014; Safiri et al. 2022). Key risk factors for CHD encompass age, gender, smoking, obesity, hypertension, hyperlipidemia, diabetes, family history, etc. (Jousilahti et al. 1999). Among these, diet is increasingly recognized as a key modifiable factor for cardiovascular prevention (Lichtenstein et al. 2021).

Breakfast, often considered the most crucial meal, has been extensively studied for its role in establishing the metabolic baseline that influences energy balance and nutrient allocation throughout daily activities (Betts et al. 2014; Chowdhury et al. 2016). Observational evidence suggests that skipping breakfast is linked to an increased risk of cardiovascular diseases (Cahill et al. 2013; Ofori‐Asenso et al. 2019). Nonetheless, observational studies are vulnerable to confounding and reverse causation, making it uncertain whether the observed associations reflect true causal effects (Smith et al. 2008). Thus, further investigation with methods that can address causality is warranted.

Mendelian randomization (MR) analysis is a novel approach for assessing causality, in which genetic variants—commonly single nucleotide polymorphisms (SNPs)—serve as instrumental variables (IVs) for exposures (Gupta et al. 2017). Because alleles are randomly allocated at conception, MR resembles a “natural” randomized controlled trial and is less affected by confounding or reverse causation (Swanson et al. 2017). With the increasing availability of genome‐wide association study (GWAS) summary statistics, MR has been widely applied to investigate modifiable risk factors for cardiovascular diseases (Ding et al. 2024; Weng et al. 2023). Here, we aimed to perform a comprehensive two‐sample MR study to determine the causal effect of breakfast skipping on CHD and MI, thus emphasizing the significance of breakfast consumption, especially in individuals with cardiovascular high‐risk factors.

## 2 Materials and Methods

### 2.1 Study Design

**FIGURE 1:**
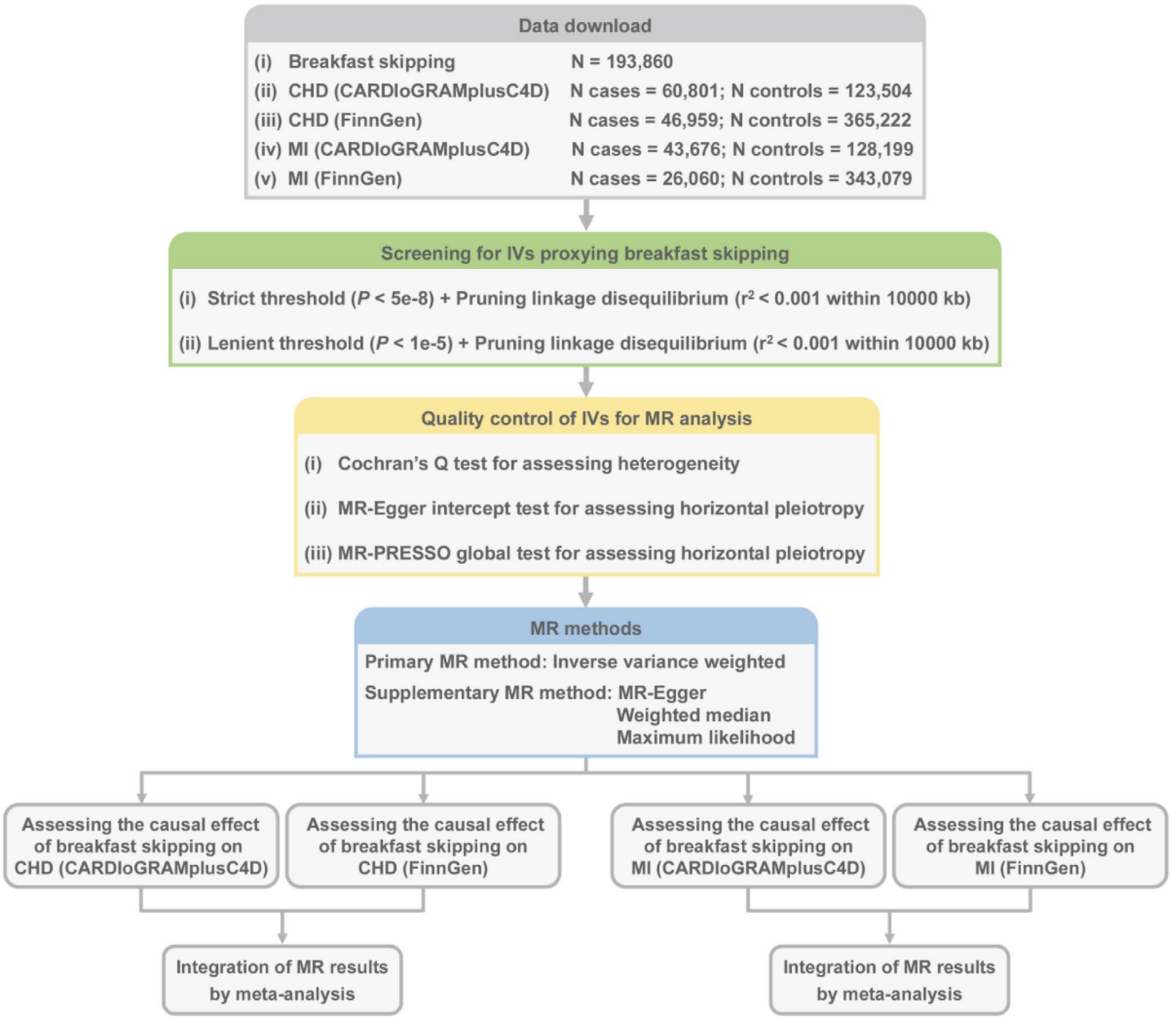
General process of the present study.

Figure 1 shows the procedure of the present MR study. This study was conducted based on the framework of two‐sample MR analysis. Specifically, summary statistics for each phenotype were obtained from publicly available databases that store GWAS summary statistics. Subsequently, IVs proxying for breakfast skipping were screened based on two sets of thresholds, which were followed by quality controls. Next, the causal effect of breakfast skipping on CHD and MI in the two diverse cohorts was assessed based on the IVs. Finally, MR estimates in both cohorts were integrated by meta‐analysis. Detailed descriptions of each step are provided in the subsequent sections. Since all data were obtained from publicly accessible databases, secondary ethical approval and informed consent were not required.

### 2.2 GWAS Summary Statistics for Breakfast Skipping

Table 1 shows the details of all GWAS summary statistics used for this MR analysis. The GWAS summary‐level statistics for breakfast skipping were derived from a large‐scale GWAS conducted by Dashti et al., including a total of 193,860 participants of European ancestry from the UK Biobank cohort (Dashti et al. 2019). Breakfast skipping was assessed from up to five recalls collected with the Oxford WebQ (a web‐based 24‐h diet recall tool) and categorized as “breakfast skipping” (always answered “No”), “sometimes breakfast skipping” (sometimes answered “Yes”), and “breakfast consumers” (always answered “Yes”). Ultimately, the GWAS identified that 25%, 20%, and 55% of participants were classified as always, sometimes, and never skipping breakfast, respectively. Summary statistics for breakfast skipping are publicly available through https://sleep.hugeamp.org/downloads.html.

**TABLE 1:** Details of GWAS summary statistics included in the present MR study.

Type	Traits	Data source	Samplesize	Data download
Exposure	Breakfast skipping	Dashti et al. (2019)	193,860	https://sleep.hugeamp.org/downloads.html
Outcome	Coronary heart disease	CARDIoGRAMplusC4D	Number of cases: 60,801 Number of controls: 123,504	https://gwas.mrcieu.ac.uk/datasets/ieu‐a‐7/
		FinnGen	Number of cases: 46,959 Number of controls: 365,222	https://r10.finngen.fi/
	Myocardial infarction	CARDIoGRAMplusC4D	Number of cases: 43,676 Number of controls: 128,199	https://gwas.mrcieu.ac.uk/datasets/ieu‐a‐798/
		FinnGen	Number of cases: 26,060 Number of controls: 343,079	https://r10.finngen.fi/

### 2.3 GWAS Summary Statistics for CHD and MI

To enhance the reliability of the analysis, summary statistics from two distinct cohorts for CHD and MI were utilized in this MR study. In the CARDIoGRAMplusC4D consortium, there are a total of 60,801 cases of CHD and 123,504 controls, as well as 43,676 cases of MI and 128,199 controls. Additionally, in the FinnGen R10 cohort, there are 46,959 cases of CHD and 365,222 controls, as well as 26,060 cases of MI and 343,079 controls. The summary statistics derived from the CARDIoGRAMplusC4D consortium were obtained from the IEU OpenGWAS database (https://gwas.mrcieu.ac.uk/) (Elsworth et al. 2020). The summary statistics from FinnGen were obtained from the FinnGen 10th release data (https://r10.finngen.fi/) (Kurki et al. 2023).

### 2.4 Statistical Analysis

Based on two different screening thresholds, namely a strict threshold of *p* < 5e−8 and a lenient threshold of *p* < 1e−5, SNPs were filtered from the GWAS summary statistics of breakfast skipping. Subsequently, to ensure the independence of IVs, SNPs in linkage disequilibrium were excluded (*r* ^2^ > 0.001 within 10,000 kb) (Hemani et al. 2018). Next, the effect sizes, standard errors, and *p*‐values of the corresponding IVs were extracted from the GWAS summary statistics of CHD and MI. Finally, the effect allele and other allele of IVs for the exposure (breakfast skipping) and outcome (CHD or MI) were harmonized, and the data were merged. SNPs potentially associated with the outcomes (*p* < 0.01) were excluded to eliminate potential horizontal pleiotropy. The *F*‐statistics for all IVs were calculated, and only IVs with *F*‐statistics greater than 10 were included to ensure that the MR analysis was not biased by weak instruments (Pierce et al. 2011).

Furthermore, multiple methods were employed for quality control to ensure the reliability of IVs. Cochran's *Q* test was used to assess heterogeneity, specifically the differences in MR estimates among different individual IVs. Both the MR‐Egger intercept test and the MR‐PRESSO global test were used to assess the horizontal pleiotropy of IVs, that is, the extent to which IVs affect CHD or MI via pathways other than breakfast skipping.

Following quality control procedures, formal MR analyses were conducted to assess the causal effects of breakfast skipping on CHD and MI. The inverse variance weighted (IVW) method serves as the primary analytical approach, conducting a meta‐analysis of causal estimates obtained from the Wald ratio method for each IV (Boehm and Zhou 2022). It is recognized as the MR method with the highest statistical power. When Cochran's *Q* test indicates no heterogeneity, the fixed‐effects IVW model is employed for meta‐integration. Conversely, in the presence of heterogeneity, the random‐effects IVW model is utilized for meta‐integration. Additionally, MR‐Egger, weighted median, and maximum likelihood methods were employed to complement the IVW results (Bowden et al. 2015, 2016). The “meta” R package was employed to perform meta‐analyses of results from two cohorts. Since the outcomes were binary variables, MR estimates were presented using odds ratios (ORs) and the corresponding 95% confidence intervals (CIs), and *p* < 0.05 was considered statistically significant. The MR results were visualized by generating a forest plot using the “forestploter” R package. The statistical power of MR estimation after meta‐analysis was calculated using the online platform (https://sb452.shinyapps.io/power/).

## 3 Results

### 3.1 Selection of IVs and Quality Control

The detailed information of IVs selected based on the strict threshold (*p* < 5e−8) and the lenient threshold (*p* < 1e−5) was respectively presented in Tables S1 and S2. All IVs exhibit robust statistical strength, with the *F*‐statistics for IVs selected based on the strict threshold ranging from 29.62 to 72.56, whereas for IVs selected based on the lenient threshold, the *F*‐statistics range from 19.60 to 72.56.

Cochran's *Q* test indicated no heterogeneity among all IVs, whether selected based on the stringent or lenient thresholds (*p* > 0.05) (Table 2). Both the MR‐Egger intercept test and the MR‐PRESSO global test indicated that IVs selected based on either the strict or lenient thresholds were not subject to significant horizontal pleiotropy (*p* > 0.05) (Table 3).

**TABLE 2:** Results of heterogeneity of IVs via the Cochran's *Q* test.

Outcome	Outcome data source	Cochran's *Q* test			
		Method	*Q*	*Q*_df	*Q*_pval
IVs screening threshold: *p* < 5e−8					
Coronary heart disease	CARDIoGRAMplusC4D	IVW	1.977	5	0.852
		MR‐Egger	1.473	4	0.831
	FinnGen	IVW	6.283	5	0.280
		MR‐Egger	3.836	4	0.429
Myocardial infarction	CARDIoGRAMplusC4D	IVW	2.566	5	0.767
		MR‐Egger	1.819	4	0.769
	FinnGen	IVW	3.816	5	0.576
		MR‐Egger	3.150	4	0.533
IVs screening threshold: *p* < 1e−5					
Coronary heart disease	CARDIoGRAMplusC4D	IVW	43.336	46	0.584
		MR‐Egger	42.476	45	0.579
	FinnGen	IVW	57.957	50	0.205
		MR‐Egger	56.597	49	0.213
Myocardial infarction	CARDIoGRAMplusC4D	IVW	52.967	47	0.255
		MR‐Egger	52.082	46	0.249
	FinnGen	IVW	61.075	51	0.158
		MR‐Egger	60.414	50	0.149

**TABLE 3:** Results of horizontal pleiotropy of IVs via the MR‐Egger intercept test and the MR‐PRESSO global test.

Outcome	Outcome data source	MR‐Egger intercept test			MR‐PRESSO global test	
		Intercept	SE	*p*	RSS obs	*p*
IVs screening threshold: *p* < 5e−8						
Coronary heart disease	CARDIoGRAMplusC4D	1.49E−02	0.021	0.517	3.148	0.831
	FinnGen	−2.37E−02	0.015	0.193	9.459	0.314
Myocardial infarction	CARDIoGRAMplusC4D	−2.01E−02	0.023	0.436	3.518	0.788
	FinnGen	−1.62E−02	0.020	0.460	5.149	0.634
IVs screening threshold: *p* < 1e−5						
Coronary heart disease	CARDIoGRAMplusC4D	5.54E−03	0.006	0.359	45.277	0.589
	FinnGen	−5.14E−03	0.005	0.283	60.648	0.204
Myocardial infarction	CARDIoGRAMplusC4D	6.21E−03	0.007	0.381	55.233	0.268
	FinnGen	4.42E−03	0.006	0.463	63.581	0.163

### 3.2 MR Results Based on IVs Screened by the Strict Thresholds

**FIGURE 2:**
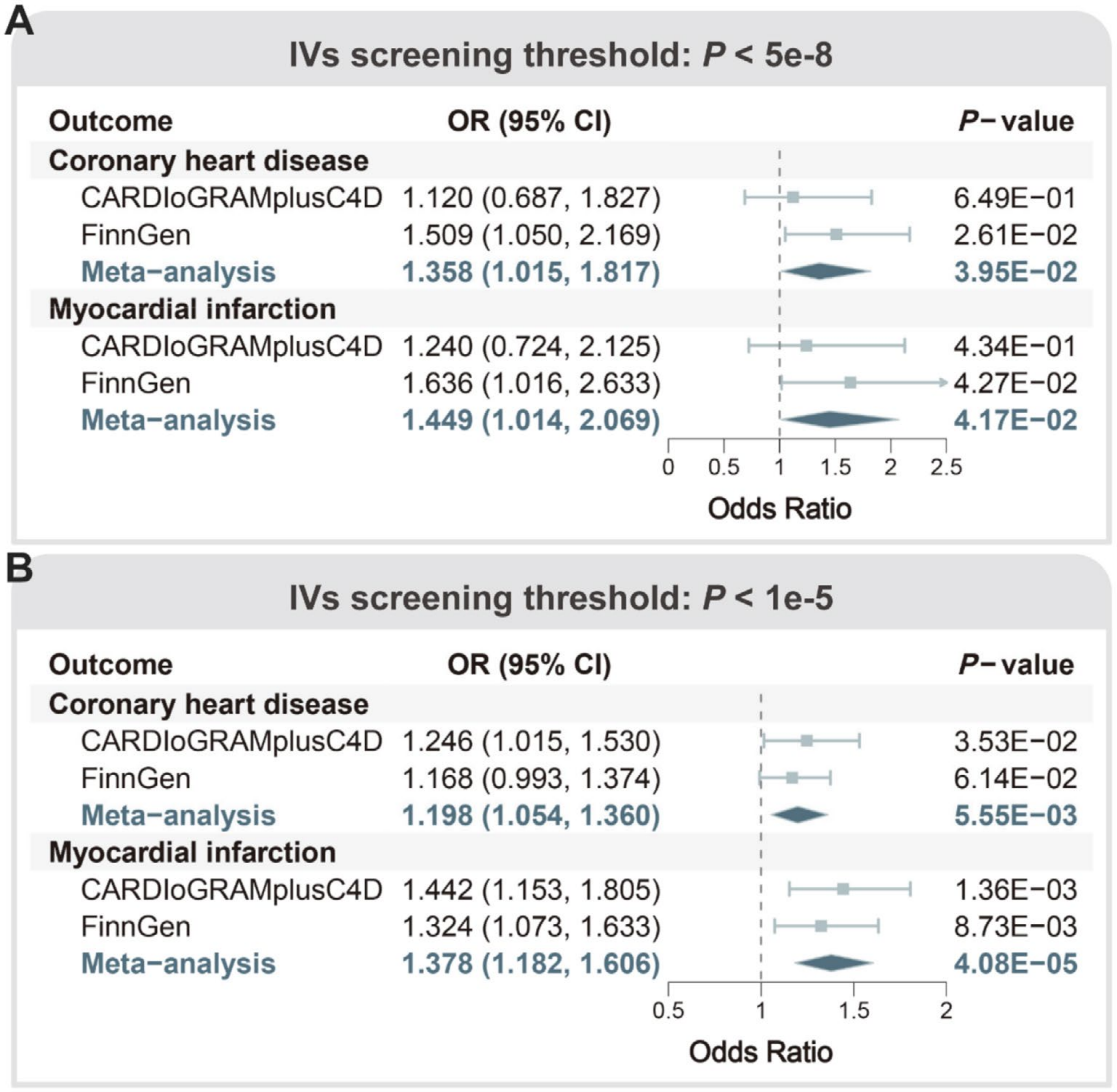
Primary MR results determined by the IVW method. (A) The causal effect of breakfast skipping on CHD and MI evaluated using the IVW method based on strict IV selection thresholds. (B) The causal effect of breakfast skipping on CHD and MI evaluated using the IVW method based on lenient IV selection thresholds.

Figure 2A shows the primary results of MR analysis using IVs screened based on the strict threshold (*p* < 5e−8). After integrating the IVW MR results from two diverse cohorts through meta‐analysis, breakfast skipping was found to significantly increase the risk of both CHD (OR = 1.358, 95% CI: 1.015–1.817, *p* = 3.95E−02) and MI (OR = 1.449, 95% CI: 1.014–2.069, *p* = 4.17E−02) simultaneously. The statistical power of the IVW estimations for CHD and MI were 88.8% and 89.1%, respectively. Additionally, the MR results obtained from MR‐Egger [(i) CHD: OR = 2.204, 95% CI: 0.692–7.018, *p* = 1.81E−01; (ii) MI: OR = 3.331, 95% CI: 0.804–13.804, *p* = 9.72E−02], weighted median [(i) CHD: OR = 1.504, 95% CI: 1.033–2.19, *p* = 3.33E−02; (ii) MI: OR = 1.575, 95% CI: 1–2.48, *p* = 5.00E−02], and maximum likelihood methods [(i) CHD: OR = 1.364, 95% CI: 1.014–1.835, *p* = 4.00E−02; (ii) MI: OR = 1.453, 95% CI: 1.013–2.085, *p* = 4.25E−02], after meta‐analysis, yielded results parallel to IVW (OR > 1) (Table S3), thereby reinforcing the adverse effects of breakfast skipping on CHD and MI.

### 3.3 MR Results Based on IVs Screened by the Lenient Thresholds

Figure 2B presents the main results of MR analysis using IVs screened based on the lenient threshold (*p* < 1e−5). Interestingly, meta‐analysis integration of IVW MR results yielded findings similar to those obtained under the strict IV selection threshold. Specifically, breakfast skipping remained significantly associated with increased risks of both CHD (OR = 1.198, 95% CI: 1.054–1.360, *p* = 5.55E−03) and MI (OR = 1.378, 95% CI: 1.182–1.606, *p* = 4.08E−05). The statistical power of the IVW estimations for CHD and MI both reached 100%. Meta‐analysis of MR results obtained from the other three MR methods, namely MR‐Egger [(i) CHD: OR = 1.271, 95% CI: 0.817–1.978, *p* = 2.87E−01; (ii) MI: OR = 1.02, 95% CI: 0.596–1.746, *p* = 9.43E−01], weighted median [(i) CHD: OR = 1.167, 95% CI: 0.97–1.404, *p* = 1.01E−01; (ii) MI: OR = 1.407, 95% CI: 1.117–1.773, *p* = 3.73E−03], and maximum likelihood methods [(i) CHD: OR = 1.204, 95% CI: 1.057–1.372, *p* = 5.22E−03; (ii) MI: OR = 1.394, 95% CI: 1.192–1.632, *p* = 3.39E−05], revealed similar findings (OR > 1) (Table S4).

## 4 Discussion

The present MR study employed various MR methods, utilized two IV selection thresholds, and performed meta‐analyses on the MR results from two cohorts, providing a comprehensive evaluation of the causal impact of breakfast skipping on the risk of CHD and MI. Overall, the MR results strongly support that breakfast skipping increases the risk of CHD and MI.

In this MR study, breakfast skipping was categorized as never, sometimes, or always. Our results indicate a dose–response relationship, with more frequent skipping linked to higher CHD and MI risks through cumulative long‐term effects. This finding is largely consistent with numerous observational studies, such as a 16‐year prospective cohort study showing that men who skipped breakfast had a 27% higher risk of developing coronary heart disease compared to those who ate breakfast (RR = 1.27, 95% CI: 1.06–1.53) (Cahill et al. 2013). Similarly, a case–control study by Sharma et al., involving 1607 Indian participants, suggests that regularly skipping breakfast significantly elevates the risk of coronary artery disease and hypertension, to a greater extent than either obesity or a sedentary lifestyle (Sharma et al. 2018). Furthermore, several meta‐analyses have also emphasized the impact of skipping breakfast on cardiovascular diseases and all‐cause mortality (Chen et al. 2020; Ofori‐Asenso et al. 2019; Takagi et al. 2019; Wang et al. 2024). Nevertheless, some studies have yielded contradictory findings. A retrospective study from a Japanese public health center showed no correlation between breakfast skipping and CHD risk (Kubota et al. 2016). Such discrepancies may partly reflect differences in study design and population characteristics. In this context, our MR approach serves as a valuable complement to observational studies, providing further evaluation of the causal association.

Multiple mechanisms can explain the potential pathways through which breakfast skipping increases the risk of CHD and MI. Studies indicate that skipping breakfast is linked to poorer diet quality among adults, with breakfast skippers often failing to meet recommended dietary allowances for vitamins and minerals and consuming higher amounts of added sugars (Deshmukh‐Taskar et al. 2010; Nicklas et al. 1998). Poor diet quality may contribute to systemic inflammation and oxidative stress, which are known to impair endothelial function and promote atherosclerosis (Hulsmans and Holvoet 2010; Zhu et al. 2021). In addition, participants who ate breakfast daily gained 1.9 kg less over 18 years compared to those who ate breakfast infrequently (0–3 days per week), indicating that regular breakfast consumption may help in controlling weight and reducing the risk of long‐term weight gain (Odegaard et al. 2013). Excess weight gain, in turn, worsens insulin resistance and elevates inflammatory markers, thereby accelerating cardiovascular damage (Battineni et al. 2021). Furthermore, cross‐sectional studies have shown a significant association between skipping breakfast and increased total cholesterol, elevated low‐density lipoprotein cholesterol levels, and decreased high‐density lipoprotein cholesterol levels in the serum, changes that may accelerate endothelial dysfunction and atherogenesis through postprandial lipid fluctuations (Deshmukh‐Taskar et al. 2013). Long‐term skipping of breakfast is also significantly associated with elevated blood pressure (Witbracht et al. 2015). Additionally, individuals who skip breakfast exhibit markers associated with impaired glucose metabolism, including elevated hemoglobin A1c, higher fasting plasma glucose, all‐day postprandial hyperglycemia, and a higher rate of impaired fasting glucose (Bi et al. 2015; Reutrakul et al. 2014). The interplay of these adverse metabolic effects—obesity, dyslipidemia, glucose instability, and hypertension—suggests a substantial cumulative impact on cardiovascular health, ultimately leading to an increased risk of CHD and MI.

This study has several advantages. Firstly, it includes two sets of CHD GWAS summary statistics and two sets of MI GWAS summary statistics, which allows for the combination of meta‐analyses within the framework of two‐sample MR analysis, thereby enhancing the statistical power of the results. Secondly, the MR studies using IVs representing breakfast skipping with two different thresholds yielded parallel positive results, providing reliable evidence for the harmful effects of breakfast skipping. Nevertheless, some limitations existed. Firstly, the study populations were predominantly of European descent, which may limit the generalizability of the findings to other ethnic groups. Secondly, the classification of breakfast skipping was based on self‐reported data, which may be subject to misclassification. Thirdly, MR studies can only assess the long‐term effects of exposure on outcomes and are unable to reflect the impacts of varying durations of exposure on outcomes.

## 5 Conclusions

Overall, based on a comprehensive MR analysis of large‐scale GWAS, the present study demonstrates that breakfast skipping significantly increases the risk of CHD and MI in European populations. Therefore, maintaining a high‐quality breakfast intake can be considered an essential primary prevention measure for CHD and MI.

## Author Contributions

**Si Cao:** conceptualization (equal), data curation (equal), formal analysis (lead), investigation (lead), methodology (lead), resources (lead), software (lead), validation (equal), visualization (equal), writing – original draft (lead), writing – review and editing (equal). **Youjie Zeng:** data curation (equal), validation (equal), visualization (equal), writing – review and editing (equal). **Gong Chen:** conceptualization (equal), funding acquisition (lead), project administration (lead), supervision (lead), writing – review and editing (equal).

## Ethics Statement

This study was performed based on publicly available GWAS summary statistics, and therefore no additional ethical approval or informed consent was required.

## Supporting information


**Table S1:** Details of IVs for MR analysis assessing the causal effect of breakfast skipping on coronary heart disease and myocardial infarction (IVs screening threshold *p* < 5e−8).
**Table S2:** Details of IVs for MR analysis assessing the causal effect of breakfast skipping on coronary heart disease and myocardial infarction (IVs screening threshold *p* < 1e−5).
**Table S3:** Results of MR analysis assessing the causal effect of breakfast skipping on coronary heart disease and myocardial infarction (IVs screening threshold *p* < 5e−8).
**Table S4:** Results of MR analysis assessing the causal effect of breakfast skipping on coronary heart disease and myocardial infarction (IVs screening threshold *p* < 1e−5).

## Data Availability

The data used for this MR study is shown in Table 1. The analysis code supporting the findings of this study is openly available in Figshare at https://doi.org/10.6084/m9.figshare.30027304.
